# Female Patients With Mucopolysaccharidosis II (MPS II): Insights From the Hunter Outcome Survey

**DOI:** 10.1002/jmd2.70046

**Published:** 2025-12-02

**Authors:** Barbara K. Burton, Hernan Amartino, Roberto Giugliani, Christoph Kampmann, Julian Raiman, Maurizio Scarpa, Anna Tylki‐Szymańska, Jennifer Audi, Jaco Botha, Siddarth Jain, Joseph Muenzer

**Affiliations:** ^1^ Ann & Robert H. Lurie Children's Hospital of Chicago Northwestern University Chicago Illinois USA; ^2^ Hospital Universitario Austral Buenos Aires Argentina; ^3^ Department of Genetics/UFRGS Medical Genetics Service/HCPA, INAGEMP and Casa dos Raros Porto Alegre Brazil; ^4^ Dasa Genômica São Paulo Brazil; ^5^ Johannes Gutenberg University Mainz Germany; ^6^ Paediatric Inherited Metabolic Diseases Department, Birmingham Children's Hospital Birmingham Women's and Children's NHS Foundation Trust Birmingham UK; ^7^ Udine University Hospital Udine Italy; ^8^ Children's Memorial Health Institute Warsaw Poland; ^9^ Takeda Pharmaceuticals International AG Zurich Switzerland; ^10^ Takeda Development Center Americas Inc. Cambridge Massachusetts USA; ^11^ University of North Carolina at Chapel Hill Chapel Hill, North Carolina USA

**Keywords:** enzyme replacement therapy, Hunter Outcome Survey, Hunter syndrome, idursulfase, mucopolysaccharidosis type II, X‐linked inheritance

## Abstract

Mucopolysaccharidosis II is a rare, X‐linked disease, with very few reports of affected female patients. Natural history data describe a predominantly male population, and appropriate disease characterization in female patients is lacking. This analysis explores the somatic disease burden and clinical progression of female patients with MPS II enrolled in the Hunter Outcome Survey (HOS; NCT03292887), a global disease registry. In total, 15 female patients were identified, representing 1.1% of the total patients in HOS. The median ages at first symptom onset and diagnosis were 1.8 and 3.1 years, respectively. A total of 8/14 (57.1%) of patients had cognitive impairment at the latest visit. X‐chromosome abnormalities were reported in two patients. Most patients (11/15, 73.3%) had received at least one dose of idursulfase, which was generally well tolerated; no serious adverse events during follow‐up were considered treatment‐related. Musculoskeletal and ear symptoms were present in all 14 patients with data recorded. Almost all females also experienced neurological, abdominal/gastrointestinal, and pulmonary disease, similar to the symptomatology reported in males. Most patients underwent surgery (41 procedures in 12 patients). Two participants had a male sibling with MPS II who was also enrolled in HOS. Both sibling sets had missense variants and demonstrated several differences in signs/symptoms between the male and female siblings. Notably, only the female siblings displayed cognitive impairment. This report illustrates the disease burden in female patients with MPS II, helping to inform clinicians about the likely prognosis for this extremely rare subgroup of patients.

**Summary**

- Registry data from the Hunter Outcome Survey, which represents the largest sample of female patients with MPS II to date, illustrate the high disease burden in this population and similarities to observations in male patients with MPS II.

## 1 Introduction

Mucopolysaccharidosis II (MPS II; Hunter syndrome; OMIM 309900) is a lysosomal storage disease caused by deficient activity of iduronate‐2‐sulfatase (I2S), resulting in accumulation of glycosaminoglycans (GAGs) throughout the body [1]. The reported global incidence of MPS II is 0.38–2.16 per 100 000 live births [2]. MPS II is an X‐linked disorder, but rare female cases have been reported [3, 4, 5, 6, 7, 8, 9, 10].

Existing MPS II literature reports a wide range of signs and symptoms from the predominantly male population, including coarse facial features, hepatosplenomegaly, respiratory disease, cardiovascular disease, and joint stiffness [2, 11, 12, 13]. About two‐thirds of patients also have central nervous system involvement (neuronopathic disease) and cognitive impairment [14, 15].

In female patients, MPS II can arise from heterozygous variants in the gene encoding iduronate‐2‐sulfatase (*IDS*), owing to chromosomal translocations, X‐chromosome monosomy, or, most frequently, skewed X‐inactivation [3]. Several phenotypes are possible depending on the extent of skewed X‐inactivation [7]. Approximately 18 cases of female patients are recorded in the literature to date (two of whom we believe are included in the present study) [3], with no prevailing *IDS* variants (Table S1) [4, 5, 6, 7, 8, 9, 10, 16]. Typically, case reports describe a high disease burden for female patients [4, 6, 7, 8, 9, 10].

Treatment for MPS II is available with the enzyme replacement therapy (ERT) intravenous recombinant I2S (idursulfase [Elaprase], Takeda Pharmaceuticals U.S.A. Inc., Lexington, MA, USA) [17, 18]. Idursulfase has been shown to stabilize or improve somatic manifestations of MPS II in male patients [19, 20, 21, 22, 23].

HOS (NCT03292887; funded by Takeda Pharmaceuticals International AG) was a large, multicenter, observational registry that collected long‐term data on the natural history of MPS II and the treatment of patients with intravenous idursulfase [11]. Building on published case reports [4, 5, 6, 7, 8, 9, 10], we explore disease burden in 15 female patients with MPS II.

## 2 Methods

### 2.1 HOS Registry Design

HOS collected data from patients with MPS II who were either untreated or received treatment with intravenous idursulfase and who were alive at enrollment (prospective patients) or deceased before enrollment (retrospective patients). Data types collected in HOS have been reported previously [11, 24].

Independent review board/ethics committee approval was obtained for all participating centers before patient enrollment. Written informed consent was obtained from patients, their parents, or a legal representative.

### 2.2 Patient Population

This analysis included all female patients enrolled in HOS (treated and untreated, prospective and retrospective) that provided informed consent. Countries of origin are omitted to protect patient anonymity. Female patients were required to have a confirmed biochemical or genetic diagnosis of MPS II.

### 2.3 Statistical Analyses

Available demographic data, ERT status, disease characteristics, surgeries, safety data, and causes of death, if relevant, were summarized for all patients (final locked database cut off: September 26, 2023). Treated patients were defined as those who had received at least one dose of ERT. Descriptive analyses were performed using medians and 10th–90th percentiles (P10, P90), unless stated otherwise.

Data on key clinical and biochemical parameters were plotted against age; figures show individual patient profiles. Surgeries were classified as one of 13 predefined categories, including “other.” Free‐text fields in “other” were inspected and, if required, recategorized (Table S2).

Safety analyses included the number of adverse events (AEs) and serious AEs (SAEs), including specification of those that were drug‐related, infusion‐related, or severe. Infusion‐related events are drug‐related events with onset during or within 24 h of the infusion.

## 3 Results

### 3.1 Patient Characteristics and Demographics

This analysis included 15 female patients with MPS II (Table 1) from 10 countries across Europe, North America, and Latin America, representing 1.1% of all patients enrolled in the HOS registry (*N* = 1332).

**TABLE 1:** Demographics and background characteristics.

		Treated (*n* = 11)	Untreated (*n* = 4)	Overall (*N* = 15)
Status at enrollment	Retrospective^a^	0 (0.0)	3 (75.0)	3 (20.0)
	Prospective	11 (100.0)	1 (25.0)	12 (80.0)
Region	Europe	8 (72.7)	2 (50.0)	10 (66.7)
	North America	2 (18.2)	1 (25.0)	3 (20.0)
	Latin America	1 (9.1)	1 (25.0)	2 (13.3)
Age at onset of symptoms, years	*n*	9	3	12
	Median (P10, P90)	2.0 (0.7, 12.0)	1.0 (0.5, 1.5)	1.8 (0.7, 4.0)
Age at diagnosis, years	*n*	9	3	12
	Median (P10, P90)	3.6 (2.3, 15.3)	1.5 (1.3, 2.0)	3.1 (1.5, 9.6)
Diagnosis before first idursulfase approval^b^	*n*	10	3	13
	Yes	5 (50.0)	3 (100.0)	8 (61.5)
	No	5 (50.0)	0 (0.0)	5 (38.5)
Median age of diagnosis in patients diagnosed before first idursulfase approval,^b^ years	*n*	5	3	8
	Median (P10, P90)	4.9 (2.3, 15.3)	1.5 (1.3, 2.0)	2.5 (1.3, 15.3)
Median age of diagnosis in patients diagnosed after first idursulfase approval,^b^ years	*n*	4	0	4
	Median (P10, P90)	3.6 (2.3, 9.6)	—	3.6 (2.3, 9.6)
Age at HOS entry, years	*n*	11	4	15
	Median (P10, P90)	10.5 (4.1, 20.0)	6.7 (4.3, 24.2)	9.5 (4.1, 21.0)
Time in HOS, months	*n*	11	4	15
	Median (P10, P90)	69.1 (10.0, 107.1)	0.0 (0.0, 0.0)	62.8 (0.0, 107.1)
Age at latest visit, years	*n*	11	1	12
	Median (P10, P90)	16.3 (11.2, 22.6)	4.8 (4.8, 4.8)	16.2 (9.5, 22.6)
Age at ERT start, years	*n*	11	0	11
	Median (P10, P90)	9.9 (4.5, 11.5)	—	9.9 (4.5, 11.5)
Age at ERT start in patients diagnosed before first idursulfase approval,^b^ years	*n*	5	0	5
	Median (P10, P90)	10.8 (8.0, 20.7)	—	10.8 (8.0, 20.7)
Age at ERT start in patients diagnosed after first idursulfase approval,^b^ years	*n*	5	0	5
	Median (P10, P90)	7.8 (4.1, 9.9)	—	7.8 (4.1, 9.9)
ERT duration, years	*n*	11	0	11
	Median (P10, P90)	6.4 (4.2, 10.7)	—	6.4 (4.2, 10.7)
Delay in ERT start, years	*n*	9	0	9
	Median (P10, P90)	5.3 (0.3, 8.5)	—	5.3 (0.3, 8.5)
Variant classification	*n*	6	2	8
	Complete deletion/large rearrangement^c^	1 (16.7)	1 (50.0)	2 (25.0)
	Nonsense^d^	0 (0)	1 (50.0)	1 (12.5)
	Missense^e^	5 (83.3)	0 (0)	5 (62.5)
Cognitive impairment at latest visit^f^	*n*	11	3	14
	Yes	7 (63.6)	1 (33.3)	8 (57.1)
	No	4 (36.4)	2 (66.7)	6 (42.9)
Deceased		0 (0.0)	3 (75.0)	3 (20.0)
Age at death, years	*n*	0	3	3
	Median (P10, P90)	—	8.5 (4.3, 24.2)	8.5 (4.3, 24.2)
Cause of death	*n*	0	3	3
	Cardiac failure	—	1 (33.3)	1 (33.3)
	Hemorrhage	—	1 (33.3)	1 (33.3)
	Respiratory failure	—	1 (33.3)	1 (33.3)
BMT surgery	Yes	0 (0.0)	1 (25.0)	1 (6.7)

*Note:* Data are *n* (%) unless otherwise stated.

Abbreviations: BMT, bone marrow transplant; ERT, enzyme replacement therapy; HOS, Hunter Outcome Survey; *IDS*, gene encoding iduronate‐2‐sulfatase; P10, 10th percentile; P90, 90th percentile.

^a^ Deceased at time of enrollment.

^b^ Idursulfase was first approved on July 24, 2006 by the US Food and Drug Administration. The European Medicines Agency approval date was January 8, 2007.

^c^ One patient had a complete deletion, and one patient had a large rearrangement.

^d^ Nonsense *IDS* variant was p.R443X.

^e^ Two patients had the missense *IDS* variant p.R468Q, and one patient each had the missense *IDS* variants p.Y523C, p.R468W, and p.R110K.

^f^ Defined based on response to the questions “Cognitive impairment? Yes/No.” This could be informed by the clinical impression of the physician or by formal cognitive tests.

The median (P10, P90) ages at first symptom onset and diagnosis were 1.8 (0.7, 4.0) and 3.1 (1.5, 9.6) years, respectively. The median age at HOS entry was 9.5 (4.1, 21.0) years; the median follow‐up time was 5.2 (0.0, 8.9) years. Patients had a median age at the latest visit of 16.2 (9.5, 22.6) years; 8/14 (57.1%) patients with available data had cognitive impairment at their latest visit (seven treated and one retrospective untreated patient). For the seven treated patients with cognitive impairment, the median age at the latest visit was 12.9 (9.5, 23.7) years. In the 10 patients with genetic data, missense *IDS* variants were common (*n* = 5). Skewed X‐inactivation was detected in one patient, but no additional data were available. An *IDS* variant could not be detected in one patient; however, karyotyping revealed an X;9 chromosome translocation (q28;q12) with preferential inactivation of the normal X chromosome. It was reported that the *IDS* gene was possibly silenced by the heterochromatin region of chromosome 9. Although *I2S* activity levels are not captured in HOS, *I2S* deficiency was recorded in 11/12 patients with available information, and 5/6 patients with available data had normal activity of other sulfatases. Three patients, all retrospective (one untreated and two with missing treatment information), were reported deceased (median age at death 8.5 [4.3, 24.2] years); causes of death were cardiac failure, hemorrhage, and respiratory failure (one patient each).

Most patients (11/15, 73.3%) had received at least one dose of intravenous idursulfase; the median age at ERT start was 9.9 (4.5, 11.5) years and the median treatment duration was 6.4 (4.2, 10.7) years. The median delay in ERT start from diagnosis was 5.3 (0.3, 8.5) years; however, five of the 10 treated patients with available data were diagnosed before idursulfase was first approved. No treated patients had received a bone marrow transplant (BMT); one of the four untreated patients received a BMT at 4.3 years old, prior to HOS enrollment, and no further information is recorded.

### 3.2 Clinical Profile

In the 14 patients with symptom data, the most common classes of signs and symptoms were ear (14/14, 100%), musculoskeletal (14/14, 100%), abdominal/gastrointestinal (13/14, 92.9%), neurological (12/14, 85.7%), pulmonary (11/14, 78.6%), cardiovascular (10/14, 71.4%), mouth (10/14, 71.4%), and throat (10/14, 71.4%).

**FIGURE 1:**
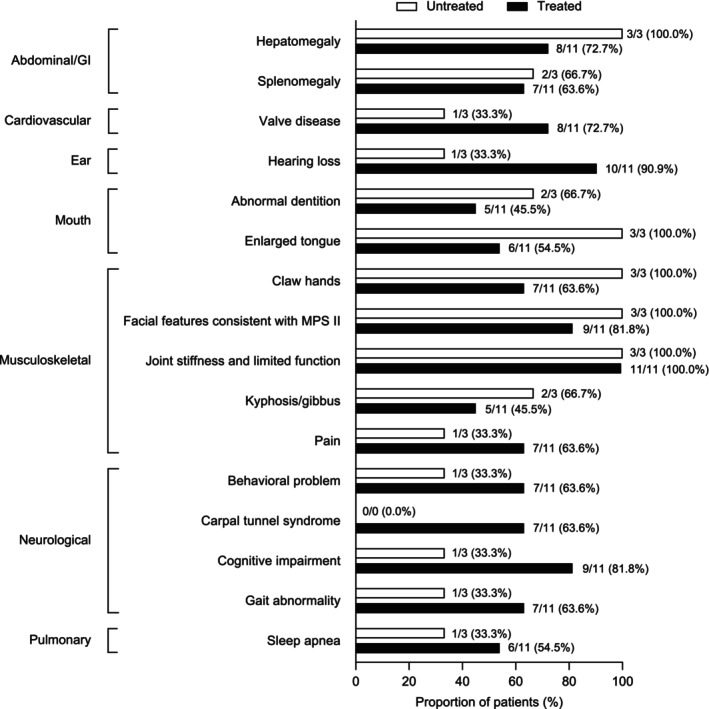
Proportion of patients displaying signs or symptoms recorded at any time by overall system organ class. Sign and symptom data were not collected for one untreated patient; an additional untreated patient was missing genitourinary and skin sign and symptom data. Only signs and symptoms shown in ≥ 50% of patients of the total sample are included in the figure. MPS II, mucopolysaccharidosis II.

Signs and symptoms reported in more than 70% of patients included joint stiffness and limited function, facial features consistent with MPS II, hearing loss, hepatomegaly, cognitive impairment, and claw hands (Table S3). Figure 1 shows the most common signs and symptoms by system organ class.

Individual profiles for uGAG levels, height, weight, and body mass index versus age are shown in Figures S1–S4. Other clinical parameters are not reported owing to a lack of available data.

### 3.3 Surgeries and Surgical Procedures

Overall, 12 patients underwent 41 surgeries/surgical procedures (Table 2). Of the 37 surgeries with available data, four were carried out in three patients before MPS II was diagnosed. The median (P10, P90) age at first surgery was 5.1 (1.3, 11.8) years; the median number of different types of surgical procedures per patient was 4 (1, 5). Three patients (20%; two treated, one untreated) had no reported surgical procedures, and 10 patients (seven treated, three untreated) had multiple types of surgery. Adenoidectomy, carpal tunnel decompression, and tonsillectomy were the most commonly reported surgeries.

**TABLE 2:** Surgeries and surgical procedures.

	Treated, *n* (%)		Untreated, *n* (%)		Overall, *n* (%)	
	Patients, (*n* = 11)	Surgeries, (*n* = 31)	Patients, (*n* = 4)	Surgeries, (*n* = 10)	Patients, (*n* = 15)	Surgeries, (*n* = 41)
Any surgery	9 (81.8)	31 (100)	3 (75.0)	10 (100)	12 (80.0)	41 (100)
Adenoidectomy	4 (36.4)	4 (12.9)	1 (25.0)	1 (10.0)	5 (33.3)	5 (12.2)
Carpal tunnel decompression	5 (45.5)	5 (16.1)	0 (0.0)	0 (0.0)	5 (33.3)	5 (12.2)
Tonsillectomy	3 (27.3)	3 (9.7)	2 (50.0)	2 (20.0)	5 (33.3)	5 (12.2)
Port‐a‐cath placement/replacement	4 (36.4)	4 (12.9)	0 (0.0)	0 (0.0)	4 (26.7)	4 (9.8)
Ear tube insertion	3 (27.3)	3 (9.7)	1 (25.0)	1 (10.0)	4 (26.7)	4 (9.8)
Hernia repair	1 (9.1)	1 (3.2)	2 (50.0)	2 (20.0)	3 (20.0)	3 (7.3)
Dental	2 (18.2)	2 (6.5)	0 (0.0)	0 (0.0)	2 (13.3)	2 (4.9)
Gastrostomy/PEG tube insertion	1 (9.1)	1 (3.2)	1 (25.0)	1 (10.0)	2 (13.3)	2 (4.9)
Tracheotomy	0 (0.0)	0 (0.0)	2 (50.0)	2 (20.0)	2 (13.3)	2 (4.9)
Femoral osteotomy	1 (9.1)	1 (3.2)	0 (0.0)	0 (0.0)	1 (6.7)	1 (2.4)
Intracranial shunt placement/replacement	1 (9.1)	1 (3.2)	0 (0.0)	0 (0.0)	1 (6.7)	1 (2.4)
Other^a^	3 (27.3)	5 (16.1)	1 (25.0)	1 (10.0)	4 (26.7)	6 (14.6)
Unknown	1 (9.1)	1 (3.2)	0 (0.0)	0 (0.0)	1 (6.7)	1 (2.4)

Abbreviations: HOS, Hunter Outcome Survey; PEG, percutaneous endoscopic gastrostomy.

^a^ The “other” category includes bone marrow transplantation, eye examination under anesthesia, appendectomy, hemiepiphysiodesis bilateral of the knees, bilateral correction to valgus knees, and other knee surgery that did not fit into the predefined HOS categories. One further surgery was reported under the “other” category but recategorized into the existing “ear tube insertion” category by the HOS medical monitor based on the descriptions in the free‐text field.

### 3.4 Patients With a Brother in HOS

Two female patients had a brother with data in HOS (Table S4).

Sister 1 and her younger brother had a missense variant c.1568A>G and were diagnosed at 4.9 and 0.2 years old, with symptom onset at 2.0 and 4.0 years, respectively. The sister started ERT aged 9.9 years and her brother at 0.3 years. Treatment duration was 6.3 and 11.0 years, respectively. Both were alive at the latest visit aged 16.1 and 11.3 years. The sister experienced neurological symptoms including cognitive impairment, behavioral problems (including aggression and hyperactivity), seizure disorder, carpal tunnel syndrome, and gait abnormality, whereas her brother experienced gait abnormality without cognitive impairment. A greater number of abdominal/gastrointestinal, cardiovascular, and musculoskeletal signs/symptoms were also recorded for the sister than for the brother. Notably, the sister experienced arrhythmia, valve disease, splenomegaly, and had more joints affected by stiffness than her brother. However, unlike his sister, the brother experienced rhinorrhea, kyphosis/gibbus, enlarged adenoids, and was oxygen dependent.

Sister 2 and her older brother had a missense variant c.329G>A. The sister was aged 4.0 years at the onset of symptoms (symptom onset data missing for brother; age at diagnosis missing for both siblings). The sister started ERT aged 7.8 years and her brother at 11.5 years; both received ERT for 4.2 years. Both siblings were alive at the latest visit, aged 11.9 and 15.6 years. Both siblings experienced carpal tunnel syndrome at the latest visit, but only the sister experienced cognitive impairment; the brother underwent cervical spinal cord decompression. The sister had more musculoskeletal signs/symptoms than her brother, whereas the brother, but not the sister, experienced hepatosplenomegaly, hernias, heart failure, and murmurs; both siblings had valve disease.

### 3.5 Safety

Of the 11 treated patients, five experienced at least one AE and two experienced one or more infusion‐related AEs (Table S5). The most common system organ classes of AEs in treated patients were nervous system disorders, investigations, and infections and infestations. Infusion‐related reactions included increased body temperature (three events in one patient) and headache (one event in one patient) (Table S6).

Two patients experienced at least one SAE. One patient experienced one mild SAE (carpal tunnel syndrome). The other patient experienced eight SAEs, including cholelithiasis (mild), bronchitis, asthma, and four instances of pneumonia, all moderate in severity except for one severe case. No SAEs were considered treatment‐related (Table S5).

## 4 Discussion

To the best of our knowledge, this report from the HOS registry provides the largest analysis to date of exclusively female patients with MPS II. The 15 patients in this analysis represented 1.1% of the overall HOS patient population, in line with the known rarity of MPS II in females [3, 4, 5, 6, 7, 9, 10, 25]. The somatic disease burden was high and multiple surgeries were common, reflecting the multisystemic nature of MPS II. Cognitive impairment was reported in almost two‐thirds of this female population, which is a similar proportion to that reported for the primarily male population [11, 26].

Delineating the underlying genetic cause of MPS II in these female patients is challenging given that karyotyping was only performed in one patient with no detectable *IDS* variant. In this patient, silencing of the *IDS* gene likely resulted from an X;9 translocation and presumed preferential inactivation of the normal X chromosome. One case of complete skewed X‐activation was also reported, although it is not known if *IDS* variant testing was completed for this patient. Given the substantial disease burden observed in this cohort, it is highly likely that the patients with classified *IDS* variants also had X‐chromosome abnormalities, as reported previously [4]. Furthermore, although a confirmed diagnosis of MPS II is a requirement for HOS enrollment, the limited detail captured regarding the factors leading to an MPS II diagnosis in these female patients raises the possibility that a minority of patients may have been misdiagnosed. For example, one patient did not have a recorded *I2S* deficiency; it remains unclear whether this represents a data entry error or a misdiagnosis. One patient was also documented as having abnormal activity of other sulfatases, raising the question of whether multiple sulfatase deficiency was definitively excluded. Missing or incomplete clinical information remains an inherent limitation of registry‐based studies; nevertheless, these data highlight the importance of conducting full biochemical and genetic diagnostic evaluations for MPS II in females that present with suspected clinical symptoms of MPS II and in the females of affected families. As asymptomatic female carriers of MPS II are not expected to meet diagnostic criteria for the disease or qualify for HOS enrollment, further research is warranted to determine if female carriers may also exhibit mild MPS II symptoms.

Most patients received at least one dose of idursulfase, but ERT was typically not started until patients were almost 10 years of age, despite the median age of diagnosis being 3.6 years [26]. The reasons for delayed treatment initiation are unknown, but may reflect the limited available evidence around expected disease severity in females and challenges in accessing treatment prior to the first approval of idursulfase in July 2006. Indeed, the median age of ERT treatment initiation was higher in those diagnosed before the first idursulfase approval than in those diagnosed after (10.8 vs. 7.8 years, respectively), although the median age at diagnosis was also slightly lower (4.9 vs. 3.6 years, respectively).

The somatic manifestations of MPS II in females appear consistent with case reports of female patients [4, 6, 7, 8, 9, 10, 25], and of the general HOS population [11], with some exceptions. Genitourinary symptoms appeared less prevalent in female patients in our analysis than in the general HOS population (15% vs. ~37%), as well as heart murmur (43% vs. 62%), and umbilical hernia (43% vs. 78%) [11]; however, the latter two may be dependent on age at examination. Although age at diagnosis in this analysis was similar to that reported overall in HOS, the delay in ERT start may have contributed to the somatic disease burden seen in this population of females with MPS II [26]. In this female analysis and in previous reports of the predominantly male general HOS population, the proportion of patients with MPS II requiring surgical intervention was approximately 80% [26, 27]. The frequencies of ear tube insertion (27%) and hernia repair (20%) were lower in this female population than those reported from the general HOS population (51% and 50%, respectively) [27], although these differences may have arisen because of the small sample size in the present analysis.

In this female sample, the prevalence of neurological signs and symptoms, such as cognitive impairment, behavioral problems, and gait abnormalities, was similar to that recorded in the general HOS population [11, 26]. In the two sibling comparisons, the sisters but not the brothers presented with cognitive impairment. The reason for this is unclear, but it is possible that X‐chromosome abnormalities not captured in HOS were present in the female siblings resulting in differential disease presentation (e.g., differences in heparan storage). To better understand the pathophysiology of cognitive impairment, a karyotype and microarray along with DNA sequencing should at least be performed on all female individuals with MPS II.

Several somatic differences between siblings were noted, with the female siblings showing a larger constellation of symptoms than the male siblings; however, in sibling pair 1, the male sibling was diagnosed and initiated ERT substantially earlier than his sister. Although our sample size was large for females with MPS II, we nonetheless remain constrained by patient numbers and the inherent limitations of registry data, including data completeness. It was therefore not possible to analyze treatment outcomes for these patients.

Idursulfase infusions were generally well tolerated in the 11 treated patients. Nervous system disorder AEs, including headache, were the most commonly reported AEs in our analysis, whereas “infections and infestations” are the most commonly reported in the general HOS population [22]; however, these differences may be due to the small sample size in this analysis and differing follow‐up times between study populations.

Our results highlight the substantial disease burden experienced by female patients with MPS II. The prevalence of somatic manifestations and neurological involvement emphasizes the importance of early diagnosis, appropriate clinical intervention, appropriate treatment initiation, and regular monitoring of this patient population.

## 5 Conclusion

These data provide insights into the similarities and differences in the phenotype and clinical profile of male and female patients with MPS II, which, with further validation, may support more individualized patient care.

## Author Contributions

All authors made substantial contributions to the planning, analysis, and interpretation of data produced in this work. All authors contributed to the drafting and revision of the manuscript, and have read and approved the final version for submission. All authors agree to be accountable for their contributions.

## Ethics Statement

Independent Review Board/Ethics Committee approval was obtained for all participating centers. The Hunter Outcome Survey was conducted in accordance with Guidelines for Good Pharmacoepidemiology Practices, Good Research for Comparative Effectiveness principles, the Declaration of Helsinki, and the relevant principles of the International Council on Harmonisation (ICH) Good Clinical Practice guidelines (ICH E6).

## Consent

Each patient, their parents, or a legal representative provided signed and dated written informed consent for participation in HOS. All patient information is managed in accordance with national data protection standards.

## Conflicts of Interest

Barbara K. Burton has received consulting fees and/or participated in advisory boards for Agios Pharmaceuticals, Alexion, Alltrna, Amgen, Applied Therapeutics, Aro Biotherapeutics, BioMarin Pharmaceutical, Chiesi Farmaceutici, JCR Pharmaceuticals, Jnana Therapeutics, Moderna, Orchard Therapeutics, Passage Bio, Sanofi Genzyme, Takeda, Travere, and Ultragenyx. She has performed contracted research for Takeda and has been involved in company‐sponsored clinical trials with BioMarin Pharmaceutical, Denali Therapeutics, Homology Medicines, JCR Pharmaceuticals, Jnana Therapeutics, Sangamo Therapeutics, Synlogic, Takeda, and Ultragenyx. Hernan Amartino has received consulting fees, fees for service, and/or participated in advisory boards for Amicus Therapeutics, BioMarin Pharmaceutical, Janssen Pharmaceuticals, PTC Therapeutics, Sanofi Genzyme, Takeda, and Ultragenyx. He has performed contracted research for Allievex, Amicus Therapeutics, bluebird bio, JCR Pharmaceuticals, Minoryx Therapeutics, PTC Therapeutics, Sanofi Genzyme, and Takeda. Roberto Giugliani has received consulting fees, fees for service, speaker fees, and/or travel expenses from, and/or participated in advisory boards for Abeona Therapeutics, Alnylam Pharmaceuticals, Amicus Therapeutics, BioMarin Pharmaceutical, Chiesi Farmaceutici, Inventiva Pharma, Janssen Pharmaceuticals, JCR Pharmaceuticals, Novartis, Orphan Disease Center, Praxis Precision Medicines, PTC Therapeutics, REGENXBIO, Sanofi Genzyme, Sigilon Therapeutics, Swedish Orphan Biovitrum, Takeda, and Ultragenyx. He has performed contracted research for or received research grants from Allievex, Amicus Therapeutics, BioMarin Pharmaceutical, GC Biopharma, Idorsia, Janssen Pharmaceuticals, JCR Pharmaceuticals, Lysogene, Sanofi Genzyme, Takeda, and Ultragenyx. Christoph Kampmann has received consulting and/or other fees from and/or has participated in advisory boards for BioMarin Pharmaceutical, Sanofi Genzyme, Gore, and Takeda. Julian Raiman has received consulting and/or speaker fees from and/or has participated in advisory boards for BioMarin Pharmaceutical, Sanofi Genzyme, and Shire (a Takeda company). He has been involved in company‐sponsored clinical trials with BioMarin Pharmaceutical, Denali Therapeutics, Shire (a Takeda company), Sanofi Genzyme, and Cyclo Therapeutics. Maurizio Scarpa has received consulting fees, fees for service, and/or participated in advisory boards for Actelion Pharmaceuticals, Alexion Pharmaceuticals, BioMarin Pharmaceutical, Chiesi Farmaceutici, Orchard Therapeutics, Orphazyme, Paradigm Biopharmaceuticals, PTC Therapeutics, Sanofi Genzyme, Takeda, and Ultragenyx. He has performed contracted research for Alexion Pharmaceuticals, BioMarin Pharmaceutical, CTD Pharma, Orchard Therapeutics, Orphazyme, Paradigm Biopharmaceuticals, PTC Therapeutics, Sanofi Genzyme, and Takeda. Anna Tylki‐Szymańska has received consulting and/or other fees from and/or has participated in advisory boards for Alexion, AstraZeneca, BioMarin Pharmaceutical, Chiesi, Orphazyme, Sanofi Genzyme, Synageva BioPharma, and Takeda. Jennifer Audi was an employee of Takeda and a stockholder of Takeda Pharmaceutical Company Limited at the time of this analysis (current affiliation is Ultragenyx Europe GmbH, Allschwil, Basel, Switzerland). Jaco Botha and Siddarth Jain are full‐time employees of Takeda and stockholders of Takeda Pharmaceutical Company Limited. Joseph Muenzer has received consulting fees and/or participated in advisory boards for Denali Therapeutics, JCR Pharmaceuticals, REGENXBIO, Sanofi Genzyme, and Takeda. He is a Principal Investigator for a post‐trial access program for intrathecal ERT for the neuronopathic form of MPS II (sponsored by Takeda), a phase 1/2 gene editing trial for adults with MPS II (sponsored by Sangamo Therapeutics), and phase 1/2 and phase 2/3 trials of intravenous ERT for MPS II (sponsored by Denali Therapeutics).

## Supporting information


**Table S1:** Genetic cause of MPS II in female patients reported in the literature.
**Table S2:** Categorization of surgeries originally listed as “other” in the HOS database.
**Table S3:** Signs and symptoms reported in more than 25% of patients.
**Table S4:** Sibling pair characteristics.
**Table S5:** Summary of overall AEs in patients who received at least one dose of ERT.
**Table S6:** Summary of AEs, SAEs, and infusion‐related reactions.
**Figure S1:** Individual uGAG profiles in treated patients (*n* = 8) with available data. Blue squares represent patients with only one data point. uGAG, urinary glycosaminoglycan.
**Figure S2:** Individual height profiles in treated patients (*n* = 11) and untreated patients (*n* = 1) with available data.
**Figure S3:** Individual weight profiles in treated patients (*n* = 11) and untreated patients (*n* = 1) with available data.
**Figure S4:** Individual body mass index profiles in treated patients (*n* = 11) and untreated patients (*n* = 1) with available data.

## Data Availability

The datasets, including the redacted study protocol, redacted statistical analysis plan, and individual participant data supporting the results reported in this article, will be made available within 3 months from initial request to researchers who provide a methodologically sound proposal. The data will be provided after its de‐identification, in compliance with applicable privacy laws, data protection, and requirements for consent and anonymization. Data requests shall follow the process described in the Data Sharing section on https://clinicaltrials.takeda.com/ and https://vivli.org/ourmember/takeda/.
