# Supplementary material for: Female Patients With Mucopolysaccharidosis II (MPS II): Insights From the Hunter Outcome Survey
Source: JIMD Rep. 2025 Dec 2;67(1):e70046. doi: 10.1002/jmd2.70046 (PMC12672032; doi:10.1002/jmd2.70046)
Supplement: Supplementary file 1 — Table S1: Genetic cause of MPS II in female patients reported in the literature. Table S2: Categorization of surgeries originally listed as “other” in the HOS database. Table S3: Signs and symptoms reported in more than 25% of patients. Table S4: Sibling pair characteristics. Table S5: Summary of overall AEs in patients who received at least one dose of ERT. Table S6: Summary of AEs, SAEs, and infusion‐related reactions. Figure S1: Individual uGAG profiles in treated patients (n = 8) with available data. Blue squares represent patients with only one data point. uGAG, urinary glycosaminoglycan. Figure S2: Individual height profiles in treated patients (n = 11) and untreated patients (n = 1) with available data. Figure S3: Individual weight profiles in treated patients (n = 11) and untreated patients (n = 1) with available data. Figure S4: Individual body mass index profiles in treated patients (n = 11) and untreated patients (n = 1) with available data. [file JMD2-67-e70046-s001.pdf]

Supplementary Material

SUPPLEMENTARY TABLE S1 Genetic cause of MPS II in female patients reported in the literature

IDS variant	X-chromosome abnormality	Reference citation
c.706–719 deletion and c.706G insertion	Complete inactivation of the nonmutant allele	Tuschl *et al* 2005[4]
p.Q293Q (synonymous mutation) with activation of a cryptic splicing site causing premature termination (28 bp deletion)	Skewed X-inactivation	Zhang *et al.* 2011[5]
p.R443* (nonsense mutation)	Skewed X-inactivation	Piña-Aguilar *et al.* 2013[6]
p.Y523C (missense mutation)	Skewed X-inactivation	Jurecka *et al.* 2012[7]
Deletion of exons 1–4 (minimal deletion range c.1–103_184)	Skewed X-inactivation	Jurecka *et al.* 2012[7]
p.Y523C (missense mutation)	Skewed X-inactivation	Kloska *et al.* 2011[8]
p.T146T (silent intragenic SNP)[a]	Balanced reciprocal X;9 translocation and skewed X-inactivation	Lonardo *et al.* 2014[9]
Intragenic inversion caused by recombination between the IDS gene and its pseudogene at intron 7	Complete skewed X-inactivation	Manara *et al.* 2010[10]

[a]Non-pathogenic alteration

Two of the patients described in the literature are thought to be enrolled in the present study.

IDS, gene encoding iduronate-2-sulfatase; MPS II, mucopolysaccharidosis II; SNP, single nucleotide polymorphism

SUPPLEMENTARY TABLE S2 Categorization of surgeries originally listed as “other” in the HOS database.

**Free-text description (*n*)**	**Categorization by the HOS medical monitor**
Knee other (1)	Other
Hemiepiphysiodesis bilateral of the knees (1)	Other
Bilateral correction to valgus knees (1)	Other
BMT (1)	Other
Eye EUA (1)	Other
Appendectomy (1)	Other
Transtympanic drainage (1)	Ear tube insertion
**Total**	**7**

BMT, bone marrow transplant; EUA, exam under anesthesia; HOS, Hunter Outcome Survey; PEG, percutaneous endoscopic gastrostomy.

SUPPLEMENTARY TABLE S3 Signs and symptoms reported in more than 25% of patients.

**Sign or symptom, *n* (%)**	**Treated (*n* = 11)**	**Untreated (*n* = 3)**	**Overall (*n* = 14)**
Joint stiffness and limited function	11 (100)	3 (100)	14 (100)
Facial features consistent with MPS II	9 (81.8)	3 (100)	12 (85.7)
Hearing loss	10 (90.9)	1 (33.3)	11 (78.6)
Hepatomegaly	8 (72.7)	3 (100)	11 (78.6)
Cognitive impairment	9 (81.8)	1 (33.3)	10 (71.4)
Claw hands	7 (63.6)	3 (100)	10 (71.4)
Valve disease	8 (72.7)	1 (33.3)	9 (64.3)
Splenomegaly	7 (63.6)	2 (66.7)	9 (64.3)
Enlarged tongue	6 (54.5)	3 (100)	9 (64.3)
Pain	7 (63.6)	1 (33.3)	8 (57.1)
Behavioral problem	7 (63.6)	1 (33.3)	8 (57.1)
Gait abnormality	7 (63.6)	1 (33.3)	8 (57.1)
Carpel tunnel syndrome	7 (63.6)	0	7 (50.0)
Sleep apnea	6 (54.5)	1 (33.3)	7 (50.0)
Abnormal dentition	5 (45.5)	2 (66.7)	7 (50.0)
Kyphosis/gibbus	5 (45.5)	2 (66.7)	7 (50.0)
Hearing aid device	6 (54.5)	0	6 (42.9)
Diarrhea	5 (45.5)	1 (33.3)	6 (42.9)
Excessive hair growth[a]	5 (45.5)	1 (50.0)	6 (46.2)
Murmur	4 (36.4)	2 (66.7)	6 (42.9)

Enlarged adenoids	4 (36.4)	2 (66.7)	6 (42.9)
Enlarged tonsils	4 (36.4)	2 (66.7)	6 (42.9)
Swallowing difficulties	4 (36.4)	2 (66.7)	6 (42.9)
Umbilical hernia	3 (27.3)	3 (100)	6 (42.9)
Acute otitis media	5 (45.5)	0	5 (35.7)
Hyperactivity	5 (45.5)	0	5 (35.7)
Murmur systolic	4 (36.4)	1 (33.3)	5 (35.7)
Seizure disorder	4 (36.4)	1 (33.3)	5 (35.7)
Abnormal reflexes	3 (27.3)	2 (66.7)	5 (35.7)
Lower airway infection/pneumonia	4 (36.4)	0	4 (28.6)
Rhinorrhea	3 (27.3)	1 (33.3)	4 (28.6)
Chronic cough/bronchitis	3 (27.3)	1 (33.3)	4 (28.6)
Restrictive/obstructive airway disease	3 (27.3)	1 (33.3)	4 (28.6)

Sign and symptom data were not collected for one untreated patient

[a]Genitourinary and skin sign and symptom data were not collected for two untreated patients.

MPS, mucopolysaccharidosis.

SUPPLEMENTARY TABLE S4 Sibling pair characteristics

	**Sibling pair 1**		**Sibling pair 2**	
	**Female sibling**	**Male sibling**	**Female sibling**	**Male sibling**
Treatment status	Treated	Treated	Treated	Treated
Age at HOS entry, years	9.5	1.6	11.9	15.6
Age at onset of symptoms, years	2.0	4.0	4.0	–
Age at diagnosis, years	4.9	0.2	–	–
Delay of diagnosis, years	2.9	0.0	–	–
Age at latest visit, years	16.1	11.3	11.9	15.6
Age at ERT start, years	9.9	0.3	7.8	11.5
ERT duration, years	6.3	11.0	4.2	4.2
Time in HOS, months	79.64	116.67	0.03[a]	0.03[a]
Cognitive problem at last visit	Yes	No	Yes	No
Deceased	No	No	No	No
IDS variant classification	c.1568A>G	c.1568A>G	c.329G>A	c.329G>A
Region	Europe	Europe	Europe	Europe

[a]Time in HOS is calculated from HOS entry to last visit date. HOS entry and last visit were on the same date for these patients.

ERT, enzyme replacement therapy; HOS, Hunter Outcome Survey; *IDS*, gene encoding iduronate-2-sulfatase.

SUPPLEMENTARY TABLE S5 Summary of overall AEs in patients who received at least one dose of ERT.

	**Treated patients, *n* (%)** **(*n* = 11)**
Any AE	5 (45.5)
Drug-related AE	2 (18.2)
Infusion-related AE	2 (18.2)
SAE	2 (18.2)
Severe AE	1 (9.1)
Serious drug-related AE	0 (0.0)
Serious severe AE	1 (9.1)

AE, adverse event; ERT, enzyme replacement therapy; SAE, serious AE.

SUPPLEMENTARY TABLE S6 Summary of AEs, SAEs and infusion-related reactions reported in more than 10% of patients treated with at least one dose of ERT listed by system organ class and preferred terms.

	**Treated, *n* (%)**	
	**Patients (*n* = 11)**	**Events**
AEs		(*n* = 32)
Any system organ class	5 (45.5)	32 (100)
Nervous system disorders	3 (27.3)	4 (12.5)
Investigations[a]	2 (18.2)	8 (25.0)
Infections and infestations[b]	2 (18.2)	7 (21.9)
Respiratory, thoracic and mediastinal disorders	2 (18.2)	6 (18.8)
Skin and subcutaneous tissue disorders	1 (9.1)	2 (6.3)
Gastrointestinal disorders	1 (9.1)	1 (3.1)
General disorders and administration site conditions	1 (9.1)	1 (3.1)
Hepatobiliary disorders	1 (9.1)	1 (3.1)
Musculoskeletal and connective tissue disorders	1 (9.1)	1 (3.1)
Vascular disorders	1 (9.1)	1 (3.1)
SAEs		(*n* = 9)
Any system organ class	2 (18.2)	9 (100)
Infections and infestations	1 (9.1)	6 (66.7)
Pneumonia	1 (9.1)	4 (44.4)
Bronchitis	1 (9.1)	1 (11.1)

Tonsillitis	1 (9.1)	1 (11.1)
Hepatobiliary disorders	1 (9.1)	1 (11.1)
Cholelithiasis	1 (9.1)	1 (11.1)
Nervous system disorders	1 (9.1)	1 (11.1)
Carpal tunnel syndrome	1 (9.1)	1 (11.1)
Respiratory, thoracic and mediastinal disorders	1 (9.1)	1 (11.1)
Asthma	1 (9.1)	1 (11.1)
Infusion-related reactions		(*n* = 4)
Any system organ class	2 (18.2)	4 (100)
Investigations	1 (9.1)	3 (75.0)
Body temperature increased	1 (9.1)	3 (75.0)
Nervous system disorders	1 (9.1)	1 (25.0)
Headache	1 (9.1)	1 (25.0)

[a]Investigations included body temperature increase, white blood cell count decrease, alanine aminotransferase increase, blood 25-hydroxycholecalciferol decrease and blood glucose increase.

[b]Infections and infestations included pneumonia, bronchitis, pharyngitis and tonsillitis.

AE, adverse event; ERT, enzyme replacement therapy; SAE, serious adverse event.

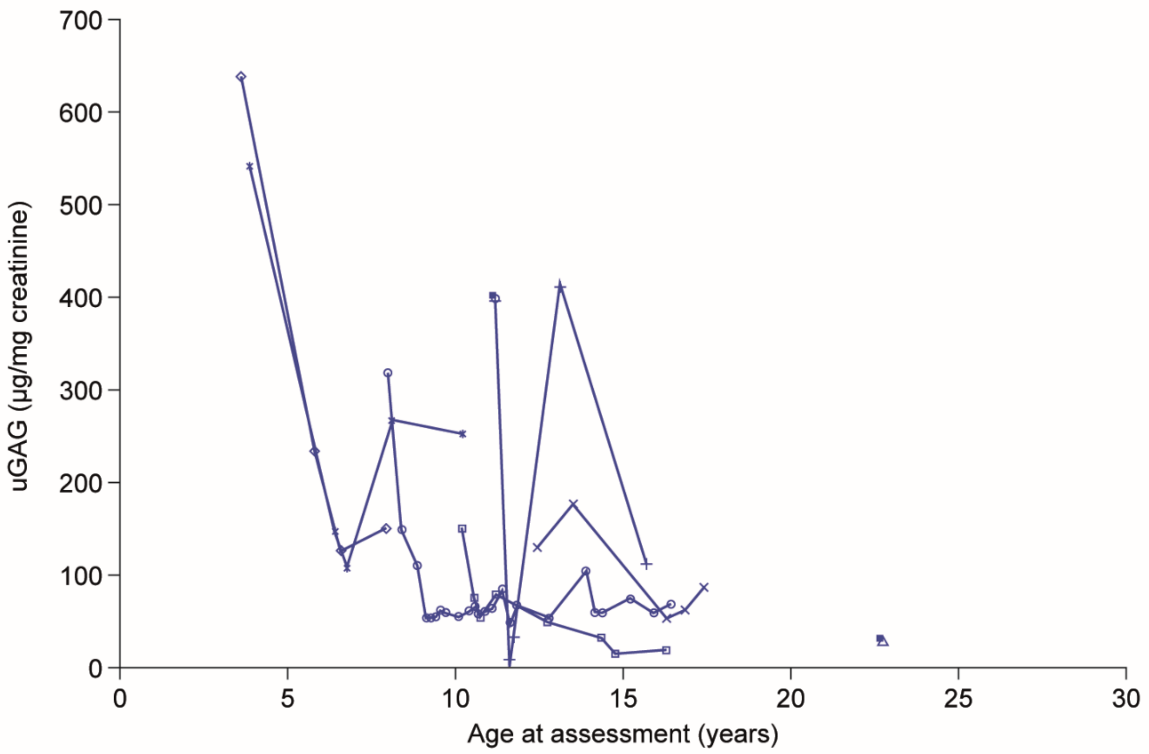

SUPPLEMENTARY FIGURE S1 Individual uGAG profiles in treated patients (*n* = 8) with available data. Blue squares represent patients with only one data point.

uGAG, urinary glycosaminoglycan.

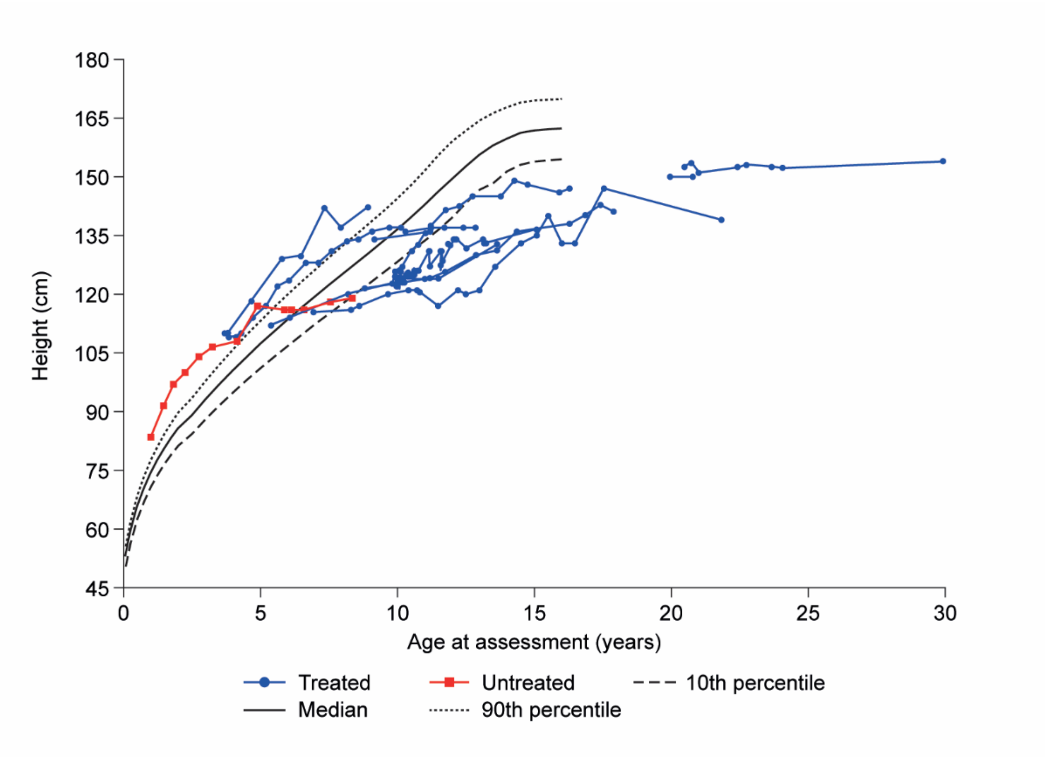

SUPPLEMENTARY FIGURE S2 Individual height profiles in treated patients (n = 11) and untreated patients (n = 1) with available data.

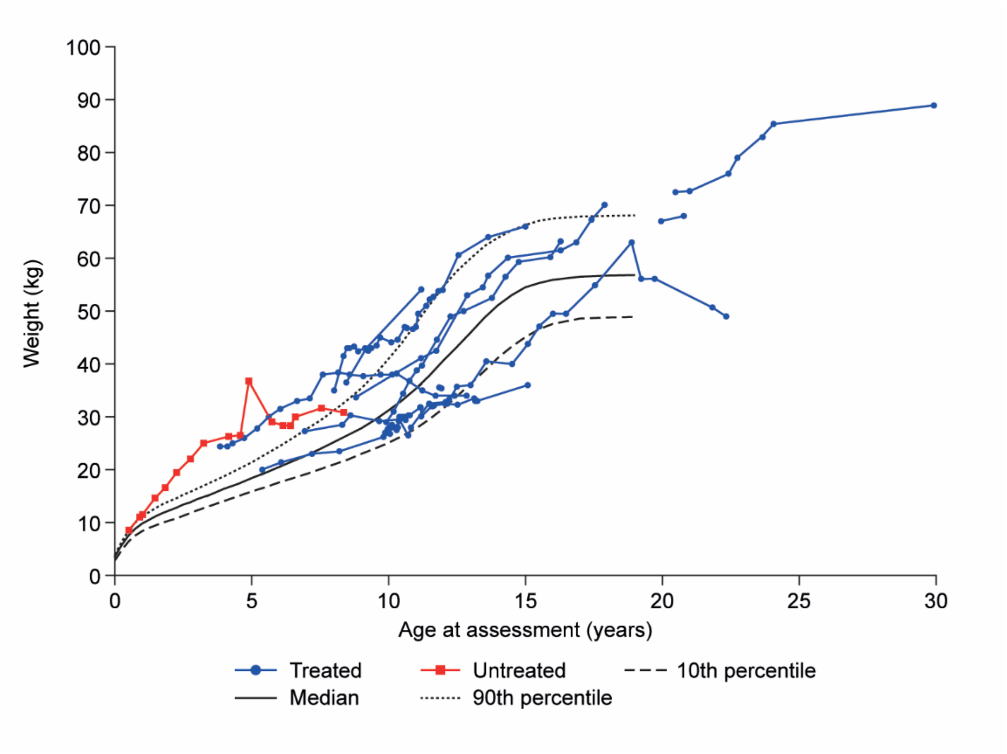

SUPPLEMENTARY FIGURE S3 Individual weight profiles in treated patients (*n* = 11) and untreated patients (*n* = 1) with available data.

Two data points presumed to be data entry errors were removed.

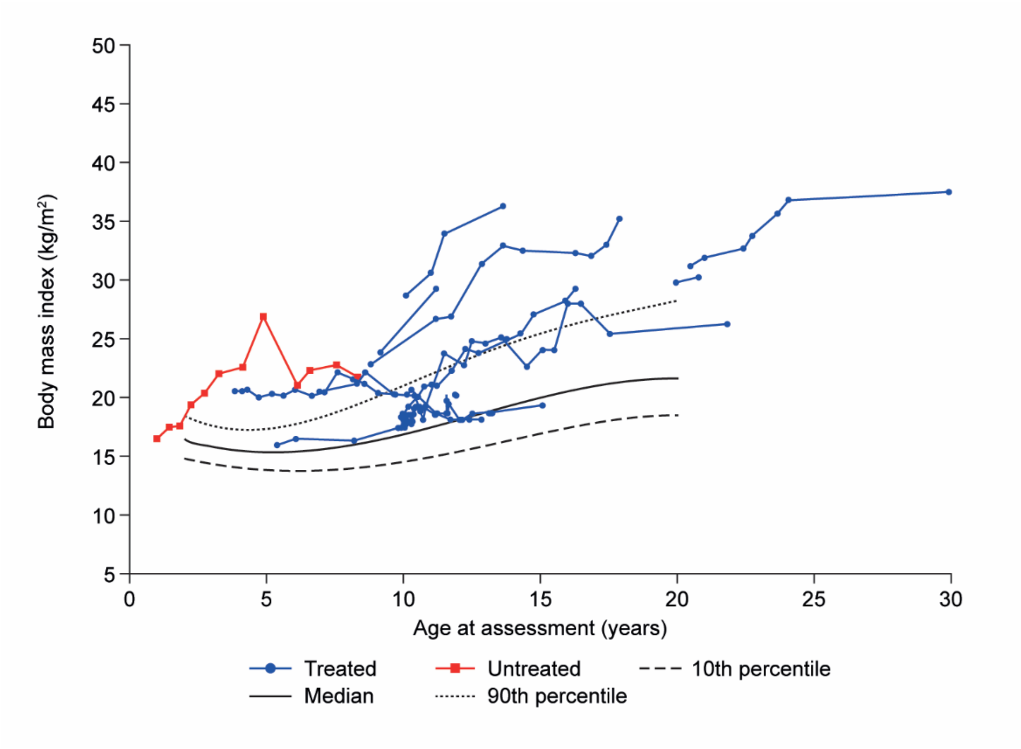

SUPPLEMENTARY FIGURE S4 Individual body mass index profiles in treated patients (n = 11) and untreated patients (n = 1) with available data.

Reference populations shown are from the Centers for Disease Control and Prevention (age 2–20 years) Z-score calculation charts. Two data points presumed to be data entry errors were removed.
